# LanCL1 promotes motor neuron survival and extends the lifespan of amyotrophic lateral sclerosis mice

**DOI:** 10.1038/s41418-019-0422-6

**Published:** 2019-09-30

**Authors:** Honglin Tan, Mina Chen, Dejiang Pang, Xiaoqiang Xia, Chongyangzi Du, Wanchun Yang, Yiyuan Cui, Chao Huang, Wanxiang Jiang, Dandan Bi, Chunyu Li, Huifang Shang, Paul F. Worley, Bo Xiao

**Affiliations:** 1 Neuroscience & Metabolism Research, State Key Laboratory of Biotherapy, West China Hospital, Sichuan University and Collaborative Innovation Center, 610041 Chengdu, PR China; 2 Laboratory of Experimental Animal Disease Model, College of Veterinary Medicine, Sichuan Agricultural University, 611130 Chengdu, PR China; 3 The Solomon H. Snyder Department of Neuroscience, Johns Hopkins University School of Medicine, Baltimore, MD 21205 USA; 4 Department of Neurology, West China Hospital, Sichuan University, 610041 Chengdu, PR China; 5 Department of Biology, Southern University of Science and Technology, 518000 Shenzhen, PR China

**Keywords:** Neuroscience, Neurological disorders

## Abstract

Amyotrophic lateral sclerosis (ALS) is a fatal neurodegenerative disease characterized by progressive loss of motor neurons. Improving neuronal survival in ALS remains a significant challenge. Previously, we identified Lanthionine synthetase C-like protein 1 (LanCL1) as a neuronal antioxidant defense gene, the genetic deletion of which causes apoptotic neurodegeneration in the brain. Here, we report in vivo data using the transgenic SOD1^G93A^ mouse model of ALS indicating that CNS-specific expression of LanCL1 transgene extends lifespan, delays disease onset, decelerates symptomatic progression, and improves motor performance of SOD1^G93A^ mice. Conversely, CNS-specific deletion of LanCL1 leads to neurodegenerative phenotypes, including motor neuron loss, neuroinflammation, and oxidative damage. Analysis reveals that LanCL1 is a positive regulator of AKT activity, and LanCL1 overexpression restores the impaired AKT activity in ALS model mice. These findings indicate that LanCL1 regulates neuronal survival through an alternative mechanism, and suggest a new therapeutic target in ALS.

## Introduction

Amyotrophic lateral sclerosis (ALS) is a devastating, rapidly progressive neurodegenerative disease characterized by the selective loss of both upper and lower motor neurons (MNs) [1]. Loss of these neurons causes muscle weakness, spasticity, atrophy, paralysis, and premature death [2]. Although the majority of cases are sporadic, ~10% cases are familial (inherited) with mutations in superoxide dismutase 1 (SOD1) being the most extensively studied [3]. Transgenic rodents carrying mutant forms of SOD1 develop a similar, progressive MN disease akin to patients [4]. ALS is more frequent in males [5], and the male patients tend to have an earlier age of onset, but the cause for this gender bias remains enigmatic [6]. Currently, Riluzole and Edaravone are the only two approved drugs for ALS clinical therapy with limited efficacy [7, 8]. Thus, how to promote neuron survival remains an unmet need in ALS treatment.

Oxidative stress has been identified as a central feature of ALS pathogenesis. A large body of studies have described evidence of increased oxidative stress in ALS patients [9–12]. Transgenic mouse based on mutant SOD1 recapitulate the oxidative damage to protein, lipid, and DNA observed in the human disease [13–15]. Although oxidative stress being a primary cause of pathogenesis in ALS or a downstream consequence of the disease has been debated, it has been proved that oxidative stress is capable of causing considerable damage to MN populations, and can also influence other mechanisms implicated in neurodegeneration in ALS [16].

MN cell death, accompanied with astrocytic and microglial cell activation, is the characteristic feature of ALS [17, 18]. Emerging evidence suggests that multiple molecular signaling pathways are involved in MN survival/death in ALS [19]. For example, in SOD1-related ALS in humans, MNs that survive the disease process show upregulation of genes that promote neuronal survival—namely, those encoding the phosphatase and tensin homolog/phosphoinositide 3-kinase/protein kinase B (PTEN/PI3K/AKT) pathway [20]. These data imply that boosting the intrinsic neuroprotective defense mechanisms may represent a promising strategy for ALS treatment.

LanCL1 (Lanthionine synthetase C-like protein 1, also known as P40 or GRP69A), is homologous to bacterial lanthionine synthetase C (LanC) family [21], which is involved in the biosynthesis of antimicrobial peptides (lantibiotics) [22]. LanCL1 was identified as a reduced glutathione (GSH)-binding protein and primarily expressed in neural tissues and testis [23–25]. In previous studies, we have revealed that LanCL1 is a critical regulator of neuronal survival during normal postnatal development and in response to oxidative stress [26]. Specifically, loss of LanCL1 leads to neuronal death, oxidative stress, and inflammation in the brain, whereas LanCL1 transgene protects neurons against exogenous peroxide-induced apoptosis [26]. Enzymatic assays reveal that LanCL1 possess ROS scavenging properties like glutathione S-transferases (GSTs) [26]. These findings prompted us to investigate whether LanCL1 could provide neuronal protection in neurodegenerative diseases, such as ALS. Interestingly, LanCL1 protein was found to be increased in the spinal cord of SOD1^G93A^ transgenic mice at presymptomatic stages [27], implicating a possible role of LanCL1 in ALS.

Here, we report that LanCL1 protects MNs against degeneration and reduces the severity of disease manifestations in the mouse model of ALS. By crossing LanCL1 conditional transgenic mice with the SOD1^G93A^ mouse model, we found that CNS-specific expression of LanCL1 transgene significantly prolongs lifespan, delays disease onset, decelerates symptomatic progression, and improves motor performance in SOD1^G93A^ mice. Reciprocally, CNS-specific deletion of LanCL1 causes neurodegenerative phenotypes, including MN loss, neuroinflammation, and oxidative damage in spinal cord. We further show that loss of LanCL1 leads to a decrease in AKT phosphorylation, whereas LanCL1 transgene restores AKT phosphorylation and mitigates oxidative stress in ALS mice. Findings suggest that LanCL1 modulates MN survival by scavenging ROS and enhancing AKT activity.

## Material and methods

### Animals

The generation and characterization of the LanCL1 conditional knockin (termed LanCL1 cKI) mice and LanCL1^flox/flox^ mice have been previously described [26]. SOD1^G93A^ [B6SJL-TgN(SOD1-G93A)1Gur] and Nestin-Cre (C57BL/6) mice were originally derived from the Jackson Laboratory (Bar Harbor, Maine, USA) and maintained on C57BL/6 and 129s4 mixed background. Male mice heterozygous for the human SOD1^G93A^ transgene were crossbred with female LanCL1^K/K^; Nestin^Cre+/−^ mice, resulting in triple-transgenic mice and control littermates. Littermates were always used as controls. The breeding strategy and the resulting genotypes are reported in Fig. 2a. Mice carrying the floxed allele of LanCL1 were crossed to Nestin-Cre transgenic mice [28] to generate LanCL1 conditional knockout (termed LanCL1 cKO) mice with LanCL1 deficiency in central nervous system (CNS). LanCL1 cKO mice were compared with WT littermates with intact LanCL1 expression. Formal methods of randomization were not used. All experiments were performed in compliance with the guidelines set forth by Sichuan University and Johns Hopkins University.

### Disease scoring and behavior analysis

**Fig. 1:**
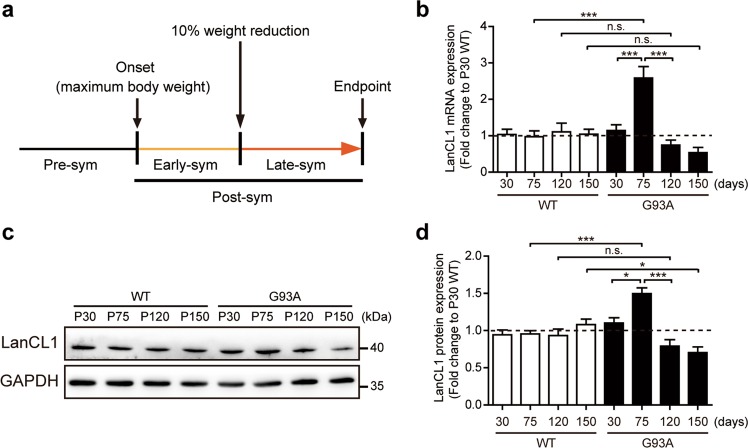
Dynamic expression of LanCL1 in the spinal cord of ALS mice. **a** Diagram depicting the definition of pathological stages of the SOD1^G93A^ mouse model of ALS. **b** Quantitative mRNA levels of LanCL1 in the lumbar spinal cord (L3–L5) of WT and SOD1^G93A^ mice at presymptomatic stage (P30 and P75), early-symptomatic stage (P120) and late-symptomatic stage (P150). Data represent mean ± SEM, *n* = 4 mice per genotype per timepoint, ***p < 0.001, n.s. nonsignificant, two-way ANOVA followed by Bonferroni post hoc test. Immunoblots (**c**) and quantifications (**d**) of LanCL1 in the lumbar spinal cord (L3–L5) of WT and SOD1^G93A^ mice at presymptomatic stage (P30 and P75), early-symptomatic stage (P120) and late-symptomatic stage (P150). Data represent mean ± SEM, *n* = 3 mice per genotype per timepoint, *p *<* 0.05, ***p *<* 0.001, n.s. nonsignificant, two-way ANOVA followed by Bonferroni post hoc test

Mice were monitored weekly throughout disease progression. Disease status was referenced to body weight changes [29]. Disease onset was defined as the age when mice reached their peak weight before weight began to decline. Disease endpoint was defined as the age at which a mouse could no longer right themselves within 30 s of being put on their side. Disease progression was defined by the duration between the onset and disease endpoint. The early-symptomatic stage (Early-sym) and late-symptomatic (Late-sym) stage was divided based on whether mice had lost 10% of their body weight. Definition of disease course analysis is reported in Fig. 1a.

Testing of motor function using a rotarod device (XR1514, Shanghai) began at 8 weeks of age. Each session consisted of three trials on the accelerating rotarod with increasing speed from 4 to 30 rpm in 5 min. The time spent on the rotarod was measured, and for all mice the average of three consecutive trials without prior training was calculated. All behavioral tests were conducted with the experimenter blind to genotypes.

### Tissue processing, immunofluorescence, and histology

Animals were deeply anesthetized with 4% chloral hydrate and were transcardially perfused with phosphate-buffered saline (PBS) followed by 4% PFA in PBS. Lumbar spinal cord (L3–L5) was isolated and post-fixed in 4% PFA overnight at 4 °C. Fixed tissues were then cryoprotected for 48 h in PBS containing 30% sucrose, embedded in O.C.T (TissueTek, Sakura) and sectioned at 20 μm using a freezing microtome (Thermo scientific HM 525). Free-floating sections were mounted onto 2% gelatin-coated slides to be processed for staining.

Frozen sections were blocked with 10% normal goat serum/1% BSA/0.3% Triton X-100 in PBS for 1 h at room temperature, and then incubated with combinations of the following primary antibodies: SMI-32 (1:1000, BioLegend, 801701), GFAP (1:1000, Merck Millipore, MAB360), Iba-1 (1:800, Wako, 019-19741). Antibodies were applied in 1% BSA/1% goat serum/0.3% Triton X-100/PBS solution for overnight at 4 °C. Sections were then briefly washed with PBS and incubated for 2 h at room temperature with appropriate combinations of secondary antibodies. After washing, sections were mounted with ProLongGold mounting medium (Life Technologies).

For Nissl staining, slides were air-dried overnight. Sections were immersed in 0.1% (w/v) cresyl violet acetate (Nissl stain) (Sigma-Aldrich) using a standard protocol, dehydrated, and coverslipped.

For each experimental group or time points, at least three animals were processed and analyzed.

### Imaging and analysis

Tissue sections were visualized using an Olympus BX63 microscope and images captured using a Photometric SenSys Olympus DP70 CCD camera and imaging software (Olympus Corporation, Tokyo, Japan). For each mouse, at least eight images (20×) were taken in the left and right ventral horn (VH) of lumbar spinal cord (L3–L5). Any image contrast and brightness adjustments were made equally between experimental groups.

To assess the extent of immunoreactivity for GFAP and Iba-1, an intensity threshold was applied to each image to include the positive labeling while minimizing the inclusion of nonspecific, background staining using ImageJ. The area occupied by the signal (thresholded pixel area) in identically sized regions of ventral spinal cord was determined for each image as previously reported [30]. Values for left and right VH were averaged for each spinal VH and 4–6 sections were analyzed for each mouse (at least three mice per genotype per age group). Results were reported as the fold change with respect to WT (P110).

### MN quantification

The number of MNs were counted in every 10th section of the ventral spinal cord in the series, i.e., at a distance of 200 μm between evaluated slices. The following criteria were used to count Nissl-stained MNs: (1) cells located in the VH, and (2) cells with a maximum diameter of 20 μm or more [31]. Likewise, for SMI-32 staining, only SMI-32^+^ cells with intact nucleus, ≥20 μm in diameter in the VH were counted [32]. Values for left and right VH that belong to the same cross-section were combined and reported as the MN number per section. The MN numbers were averaged from a minimum of 10 sections and a minimum of 140 neurons per mouse to ensure accurate sampling of heterogeneously distributed MNs. All motoneuron counts were performed in a blinded fashion.

### TUNEL staining

Mouse spinal cords were fixed as described above for the Nissl-stained sections. Lumbar spinal cord (L3–L5) sections were screened for apoptotic cells by terminal deoxynucleotidyl transferase-mediated dUTP nickend-labeling (TUNEL) method. The Apoptag^®^ Plus In Situ Apoptosis Detection kit (Roche, 11684817910) was used. All the procedures were conducted according to the protocol of the kit and of our previous study [26]. Endogenous peroxidase activity was blocked by incubation in 3% hydrogen peroxide/methanol at room temperature for 10 min. The sections were then incubated with terminal deoxynucleotidyl transferase at 37 °C for 60 min to add the digoxigenin-conjugated dUTP to the 3′-OH ends of fragmented DNA. Converter-POD (anti-fluorescein antibody, Fab fragment from sheep, conjugated with horse-radish peroxidase) was added on sample at 37 °C for 30 min. The sections were visualized using 3,3′-diaminobenzidine hydrochloride (DAB) as chromogen. Positive controls provided in the kit were simultaneously processed.

### Immunoblotting analysis

Lumbar spinal cord (L3–L5) or frontal cortex was homogenized in RIPA lysis buffer containing 50 mM Tris-HCl, (pH 7.4), 150 mM NaCl, 1% Triton X-100, 2% SDS, 1% protease inhibitor Cocktail III (Merck) and phosphatase inhibitor Cocktail III (Biovision). The lysates were centrifuged at 15,000 × *g* for 20 min at 4 °C to collect supernatants. Protein concentration was quantified using the bicinchoninic acid protein assay kit (Thermo Scientific Pierce). Proteins (20–30 μg) were electrophoresed through SDS-PAGE gels and transferred to a PVDF membrane (Merck Millipore). Membranes were blocked with 5% (w/v) skim milk in TBST for 1 h at room temperature and incubated with the primary antibodies in TBST overnight at 4 °C. The following primary antibodies were used: anti-GAPDH (1:5000, Millipore, MAB374), anti-β-Actin (1:2000, Millipore, 04-1116), anti-4-Hydroxy-2-Nonenal (4-HNE, 1:1000, Abcam, ab48506), anti-dinitrophenol (DNP, 1:1000, Millipore, S7150), anti-phospho-AKT (Ser473) (1:1000, Cell signaling, 4060S), anti-phospho-AKT (Thr308) (1:1000, Cell signaling, 2965S), anti-total AKT (1:1000, Cell signaling, 9272S), anti-BAX (1:1000, Millipore, 06-499), anti-HO-1 (1:1000, Abcam, ab13243) or anti-LanCL1 (1:1000, homemade). Anti-LanCL1 antibody was generated by immunizing rabbits with full-length GST fusion protein and affinity-purified using maltose bind protein (MBP)-tagged LanCL1 recombinant protein. Blots were washed three times in TBST for 10 min each and incubated with secondary antibodies (goat anti-rabbit IgG-HRP or goat anti-mouse IgG-HRP, 1:10,000, Thermo Pierce) for 2 h at room temperature. The proteins were detected using the ECL system and quantified using ImageJ software by taking the mean grey value of bands for target proteins normalized to GAPDH or beta actin level after subtracting background intensity. pAKT bands were normalized to the level of tAKT.

### RNA extraction and RT-PCR

Total RNAs were extracted from tissues using TRIzol reagent (Life Technologies). RNA was subjected to reverse transcription with reverse transcriptase as Manufacturer’s instructions (Thermo). Quantitative real-time PCR was performed using the Bio-Rad CFX96 system, and the relative gene expression was normalized to internal control as GAPDH. Primer sequences for SYBR Green probes of target genes are as follows: LanCL1 forward 5′-CCTTCAGGTGAACCAAGGAA-3′ and reverse 5′-CCAGAGACCTGCTTGTCCAT-3′; IL-1β forward 5′-CTGGTGTGTGACGTTCCCATTA-3′ and reverse 5′-CCGACAGCACGAGGCTTT-3′; IL-6 forward 5′-TTCCATCCAGTTGCCTTCTTG-3′ and reverse 5′-TTGGGAGTGGTATCCTCTGTGA-3′; TNFα forward 5′CATCTTCTCAAAATTCGAGTGACA-3′ and reverse 5′-TGGGAGTAGACAAGGTACAACCC-3′.

### Cell cultures and transfection

Cortical neurons from E17.5 LanCL1 wild-type (WT) and knockout mice were prepared as described previously [26]. In brief, embryos were dissected and minced well with scissors. The dissociated cells were collected by centrifugation and resuspended in Neurobasal (Gibco) medium supplemented with 5% Horse Serum (Hyclone), 2% GlutaMAX (Gibco), 2% B27 (Gibco), 100 U/ml penicillin and 100 μg/ml streptomycin (Gibco). Cells (1 × 10^6^) were plated in six-well culture plates precoated with polylysine (Sigma, 0.5 mg/ml). After 3 days in culture, 2.5 μM cytosine β-D-arabinofuranoside hydrochloride (Sigma) was added to prevent glial proliferation. Then, the neurons were subsequently maintained with Neurobasal Media (Gibco) containing 1% Horse Serum (Hyclone), 100 U/ml penicillin and 100 μg/ml streptomycin (Gibco), and 1% GlutaMAX (Gibco), 2% B27 (Gibco). These cultures contained >95% neurons and no detectable microglia. Astroglia were generated as described [33].

HEK293T and HeLa cells were obtained from the Cell Bank of China Center for Type Culture Collection (Wuhan, China). Both cell lines were authenticated by short tandem repeat profiling. Cells were cultured in Dulbecco’s high glucose modified Eagle medium (DMEM, Life Technologies) supplemented with 10% fetal bovine serum (FBS, Wisent) and 2 mM l-glutamine (Sigma). HEK293T cells were collected 48 h after transfection with Myc-pRK5 vector, LanCL1 (WT), LanCL1 (R4A), or LanCL1 (R322A) plasmid using Lipofectamine^®^ 2000 (Life Technologies), according to the manufacturer’s instructions.

### Hoechst 33342 staining

HeLa cells were transfected with 2 μg of Myc-tagged LanCL1 or Myc-pRK5 plasmid using Lipofectamine® 2000 (Life Technologies). Twenty four hours after transfection, cells were pretreated with either DMSO vehicle, 5 μM BKM120 (Selleck Chemicals, S2247) or 20 μM LY294002 (Selleck Chemicals, S1105) for 30 min prior to addition of 150 μM H_2_O_2_. Hoechst 33342 staining was performed 24 h after the H_2_O_2_ treatment as previously described [34]. Briefly, cells were washed with PBS, and then incubate the cells with 10 µg/ml Hoechst 33342 dye (Sigma-Aldrich) in the dark for 15 min at room temperature. The apoptotic cells with condensed chromatin and/or fragmented nuclei were visualized and scored under the Olympus BX63 microscope. Three coverslips were used per experimental group, with at least 1000 cells in six random fields being counted. Each experiment was repeated at least three times.

### Statistical analysis

All statistical analyses were performed using GraphPad Prism software version 6 (GraphPad software Inc). No statistical methods were used to predetermine sample sizes, but our sample sizes are similar to or greater than those reported in previous publications [35–37]. Data represent the mean and standard error of the mean (SEM). Unpaired two-tailed Student’s *t* test was used for the comparison of two means. ANOVA followed by Bonferroni’s or Tukey’s post hoc test were used for the multiple group analysis. Log-rank test was used for disease onset and survival analysis. The significance level for the two-sided analyses was set at *P* < 0.05.

## Results

### CNS-specific LanCL1 transgene expression improves the survival and motor function of ALS mice

As a first step towards the understanding of the role of LanCL1 in ALS, we characterized the temporal expression patterns of LanCL1 in SOD1^G93A^ mice. Since a decline in body weight is highly correlated with denervation-induced muscle atrophy, weight loss is well established as a simple and objective indictor of the disease course [29]. Thus, we analyzed the disease course based on body weight alterations (Fig. 1a). To evaluate LanCL1 expression at different stages of the disease, both mRNA and protein of lumbar spinal cords from SOD1^G93A^ (G93A) mice and WT littermates were analyzed at the presymptomatic stage (P30 and P75), Early-sym stage (P120) and Late-sym stage (P150). Results showed that both LanCL1 mRNA and protein levels were increased at the late presymptomatic stage (P75) as compared with WT mice (Fig. 1b–d). However, with disease development, the mRNA and protein levels of LanCL1 gradually decreased in SOD1^G93A^ spinal cord compared with WT (Fig. 1b–d).

**Fig. 2:**
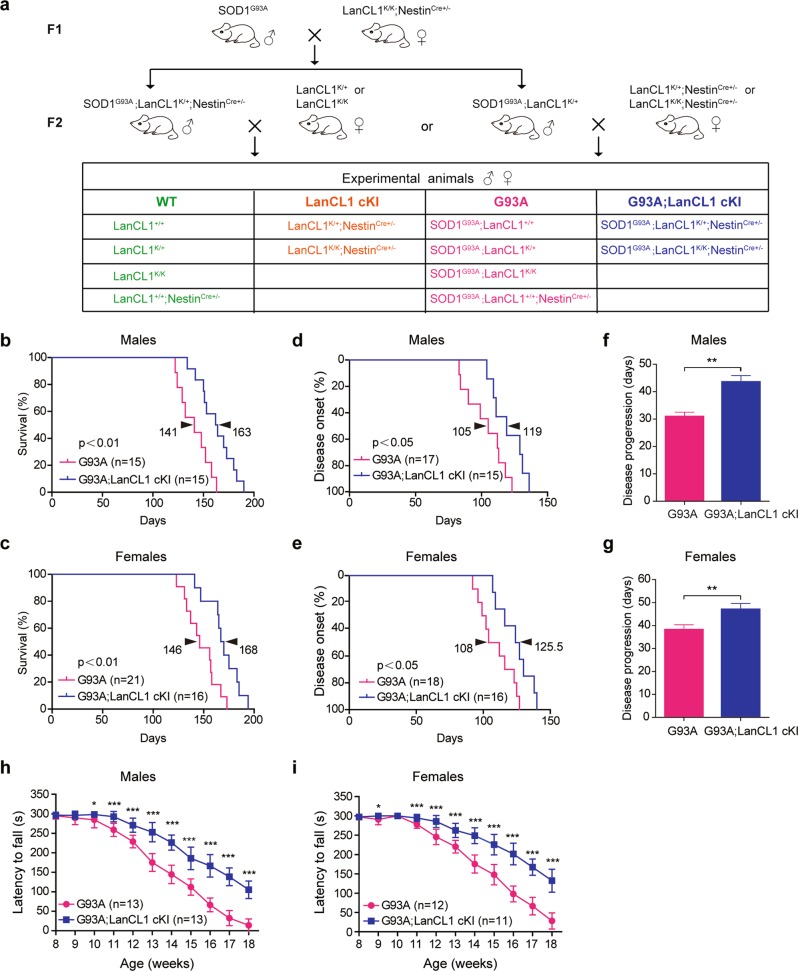
CNS-specific expression of LanCL1 transgene significantly prolongs the lifespan, delays disease onset, extends disease progression, and improves motor function of ALS mice. **a** Breeding strategy for the generation of G93A; LanCL1 cKI triple-transgenic mice and control littermates. Kaplan–Meier curves of the survival showing that LanCL1 overexpression increases median survival **b** from 141 days for G93A males (*n* = 15 mice) to 163 days for G93A; LanCL1 cKI males (*n* = 15 mice), and **c** from 146 days for G93A females (*n* = 21 mice) to 168 days for G93A; LanCL1 cKI females (*n* = 16 mice). **p *<* 0.01, by log-rank test. Kaplan–Meier curves of disease onset showing that LanCL1 overexpression delays disease onset **d** from 105 days for G93A males (*n* = 17 mice) to 119 days for G93A; LanCL1 cKI males (*n* = 15 mice), and **e** from 108 days for G93A females (*n* = 18 mice) to 125.5 days for G93A; LanCL1 cKI females (*n* = 16 mice). *p *<* 0.05, by log-rank test. Mean duration of disease (days from onset to endpoint) showing that LanCL1 overexpression extends disease progression **f** from 30.4 days for G93A males (*n* = 16 mice) to 43 days for G93A; LanCL1 cKI males (*n* = 15 mice), and **g** from 37.5 days for G93A females (*n* = 17 mice) to 46.3 days for G93A; LanCL1 cKI females (*n* = 18 mice). **p *<* 0.01, by two-tailed unpaired Student’s *t* test. Rotarod tests showing that LanCL1 overexpression improves motor function of male (**h**) and female (**i**) G93A mice. Data represent mean ± SEM, *n* = 11–13 mice per sex per genotype, *p *<* 0.05, ***p *<* 0.001, by Mann–Whitney U-tests

To examine whether LanCL1 confers neuroprotective effect in ALS mice (SOD1^G93A^), we crossed CNS-specific LanCL1 transgenic mice (LanCL1^K/K^; Nestin^Cre+/−^) with ALS mice. Four experimental groups were contrasted as follows: (1) SOD1^G93A^ mice with one or two copies of LanCL1 transgene driven by Nestin-Cre (henceforth referred to as G93A; LanCL1 cKI); (2) SOD1^G93A^ mice without LanCL1 transgene (henceforth referred to as G93A); (3) CNS-specific LanCL1 overexpression without SOD1^G93A^ (henceforth referred to as LanCL1 cKI); (4) neither LanCL1 transgene nor mutant SOD1 is expressed (henceforth referred to as WT; Fig. 2a).

We monitored the survival of G93A and G93A; LanCL1 cKI mice. In males, CNS-specific LanCL1 transgene resulted in a 22 days extension in median survival (141 days for G93A and 163 days for G93A; LanCL1 cKI, respectively; Fig. 2b). Similarly, among females, median survival was increased from 146 days for G93A mice to 168 days for G93A; LanCL1 cKI mice (*p* < 0.01) (Fig. 2c). Using peak body weight to determine disease onset [38] (Fig. 1a), we found that LanCL1 transgene delayed disease onset by ~13% (*p* < 0.05) among males, from 105 days for G93A mice to 119 days for G93A; LanCL1 cKI mice (Fig. 2d), and by ~16% (*p* < 0.05) for females, from 108 days for G93A mice to 125.5 days for G93A; LanCL1 cKI animals (Fig. 2e). In addition, LanCL1 transgene extended the progression of the disease by ~43% (*p* < 0.01) and ~24% (*p* < 0.01) for males and females, respectively (Fig. 2f, g).

Motor impairment is a prominent feature of ALS mouse model. To examine whether motor deficits in ALS mice was ameliorated by LanCL1 transgene, we analyzed the motor function by Rotarod assay. G93A; LanCL1 cKI mice exhibited better performance on Rotarod tests at all time points compared with G93A mice, beginning as early as postnatal day 63 (P63) for females and P70 for males (Fig. 2h, i). Age and gender-matched littermates confirmed that G93A; LanCL1 cKI mice were able to ambulate around their cage whereas the G93A could not (Movie 1). Behavioral tests showed that one or two copies of LanCL1 transgene did not make a significant difference. Western blotting showed that a single copy of LanCL1 transgene substantially increased LanCL1 protein level in the spinal cord relative to WT tissue, and two copies of LanCL1 did not produce additional increase in LanCL1 protein (Supplementary Fig. 1a, b).

Taken together, these results indicate that CNS-specific expression of LanCL1 transgene extends the lifespan, delays disease onset, decelerates disease progression, and improves motor function of the ALS model mouse.

### LanCL1 overexpression delays MN loss in ALS mice

**Fig. 3:**
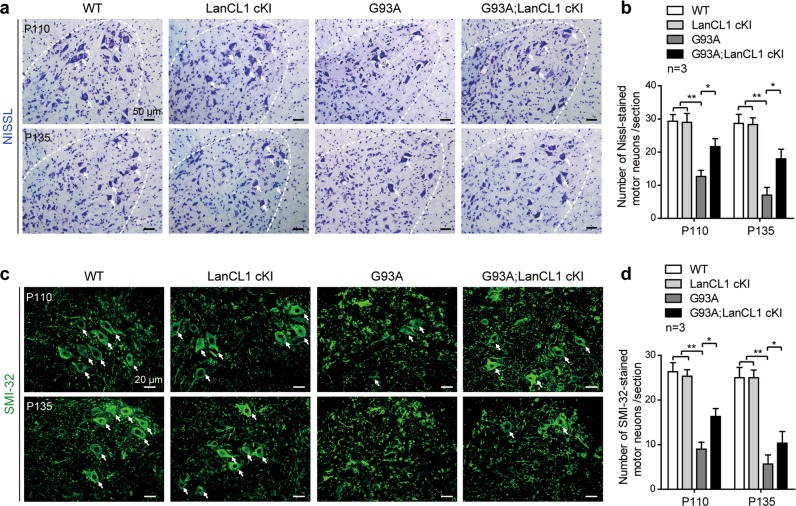
LanCL1 overexpression rescues MN loss in the spinal cord of ALS mice. Representative images (**a**) and quantifications (**b**) of Nissl-stained MNs (indicated by white arrows) in matching lumbar ventral horn (VH) at P110 and P135. Data represent mean ± SEM, *n* = 3 mice per genotype per age group, *p *<* 0.05, **p *<* 0.01, two-way ANOVA followed by Tukey’s post hoc test. Representative images (**c**) and quantifications (**d**) of SMI-32-stained alpha MNs (indicated by white arrows) in matching lumbar ventral horn at P110 and P135. Data represent mean ± SEM, *n* = 3 mice per genotype per age group, *p *<* 0.05, **p *<* 0.01, two-way ANOVA followed by Tukey’s post hoc test

The improved performance in these behavioral tests suggests that the MN degeneration in the SOD1 mutant mice might be delayed by LanCL1 transgene. To examine this notion, we compared MN survival in G93A; LanCL1 cKI and G93A mice, relative to that in WT littermates by performing Nissl staining in the spinal VH of these comparable mice at Early-sym stage (P110) and Late-sym stage (P135), respectively. Quantification of MNs with maximum diameter ≥ 20 μm showed that in G93A mice, only 47 and 33% of MNs survived at P110 and P135, respectively (Fig. 3a, b). However, in G93A; LanCL1 cKI mice, MN survival was substantially higher; 75 and 66% of the MNs survived at P110 and P135, respectively (Fig. 3a, b). The morphology and survival of MNs was further examined by performing immunostaining with the nonphosphorylated neurofilament marker—SMI-32, which is enriched in alpha MNs of the spinal cord VH [39]. Similarly, G93A mice displayed a progressive reduction in MNs in the lumbar spinal cord, whereas G93A; LanCL1 cKI mice showed significant preservation of MNs at both Early-sym stage (P110) and Late-sym stage (P135) compared with G93A mice (Fig. 3c, d). Spinal MN counts were similar between LanCL1 cKI mice and WT animals (Fig. 3a–d), indicating that no detrimental pathology occurred under CNS-specific overexpression of LanCL1 in vivo. These results indicate that LanCL1 overexpression significantly improves MN survival in the spinal cord of ALS mice.

### LanCL1 overexpression delays neuroinflammation in ALS mice

**Fig. 4:**
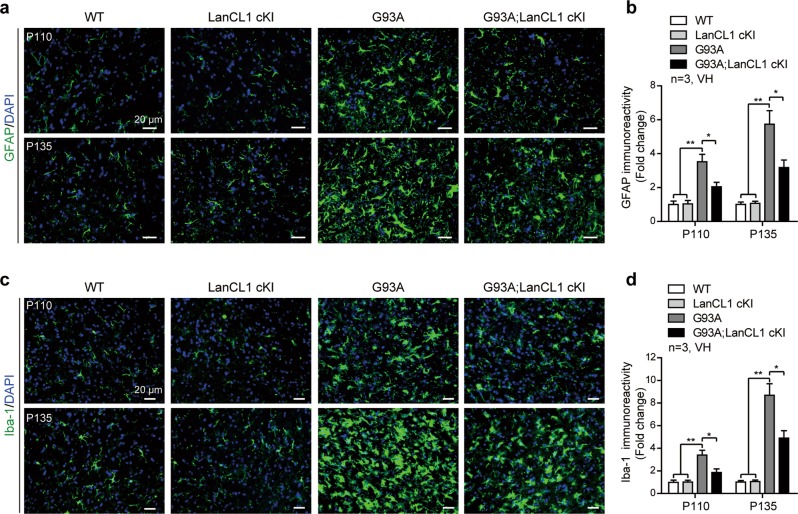
LanCL1 overexpression decreases neuroinflammation in the spinal cord of ALS mice. **a** Immunostaining of GFAP (green) and DAPI (blue) in matching lumbar ventral horn (VH) at P110 and P135. **b** Quantification of GFAP immunoreactivity in the lumbar ventral horn of indicated mice at P110 and P135. Data represent mean ± SEM, *n* = 3 mice per genotype per age group, *p *<* 0.05, **p *<* 0.01, two-way ANOVA followed by Tukey’s post hoc test. **c** Immunostaining of Iba-1 (green) and DAPI (blue) in matching lumbar ventral horn at P110 and P135. **d** Quantification of Iba-1 immunoreactivity in the lumbar ventral horn of indicated mice at P110 and P135. Data represent mean ± SEM, *n* = 3 mice per genotype per age group, *p *<* 0.05, **p *<* 0.01, two-way ANOVA followed by Tukey’s post hoc test

Neuroinflammation as indicated by astrocytic and microglial activation is a well-documented feature of ALS pathology [1]. We examined whether LanCL1 overexpression mitigates glial pathology of ALS mice. By performing immunostaining with GFAP antibody, we found that the GFAP immunoreactivity was significantly increased in the lumbar VH of G93A mice at Early-sym stage (P110, ~3.5-fold), and further increased over time, being more prominent at Late-sym stage (P135, up to ~5.7-fold) (Fig. 4a, b). However, LanCL1 overexpression decreased the immunoreactivity of GFAP by ~42% (*p* < 0.05) at P110 and by ~45% (*p* < 0.05) at P135, respectively (Fig. 4a, b). Similarly, G93A mice showed an age-dependent increase of Iba-1 immunoreactivity in the spinal VH, which was significantly reduced by LanCL1 overexpression in G93A; LanCL1 cKI littermates (Fig. 4c, d). For comparison, the immunoreactivity of GFAP and Iba-1 was not different in WT and LanCL1 cKI mice (Fig. 4a–d). These data indicate that LanCL1 overexpression delays glial pathology in the spinal cord of ALS mice.

### Deletion of LanCL1 leads to spinal cord pathology

Previously, we showed that LanCL1 KO mice develop degenerative phenotypes in the brain [26]. Here, we extend this analysis to include spinal cord of LanCL1 conditional knockout (LanCL1^f/f^; Nestin^Cre+/−^, termed LanCL1 cKO) mice. LanCL1 cKO mice exhibited a reduction of MNs by ~18% in the spinal VH at P60-P90, as indicated by Nissl staining (Supplementary Fig. 2a, b). SMI-32 staining revealed a decrease of alpha MNs in LanCL1 cKO spinal VH at the same age (Supplementary Fig. 2a, c). The loss of spinal neurons was associated with increased apoptosis, as we detected a ~3.5-fold increase in the number of TUNEL^+^ cells in the spinal VH of LanCL1 cKO mice by terminal deoxynucleotidyl transferase-mediated deoxyuridine triphosphate nickend labeling (Supplementary Fig. 2d, e). These results indicate that LanCL1 plays a role in promoting neuronal survival in spinal cord.

We next examined whether MN degeneration was accompanied by neuroinflammatory responses. The GFAP and Iba-1 immunoreactivity were significantly increased as early as P60 (Supplementary Fig. 2f–i). Consistently, the mRNA levels of pro-inflammatory cytokines, including *IL-1β*, *IL-6*, and *TNF-α* were significantly upregulated (Supplementary Fig. 2j). Additionally, LanCL1 cKO spinal cord displayed ectopic accumulation of protein carbonyl and lipid peroxidation, as indicated by DNP [40] and 4-HNE [41] western blots (Supplementary Fig. 2k–n). Consistent with neuronal damage, LanCL1 cKO mice develop late stage motor impairment, as indicated by the Rotarod tests of mice at 8–12 month (Supplementary Fig. 2o).

### LanCL1 positively regulates AKT activity

**Fig. 5:**
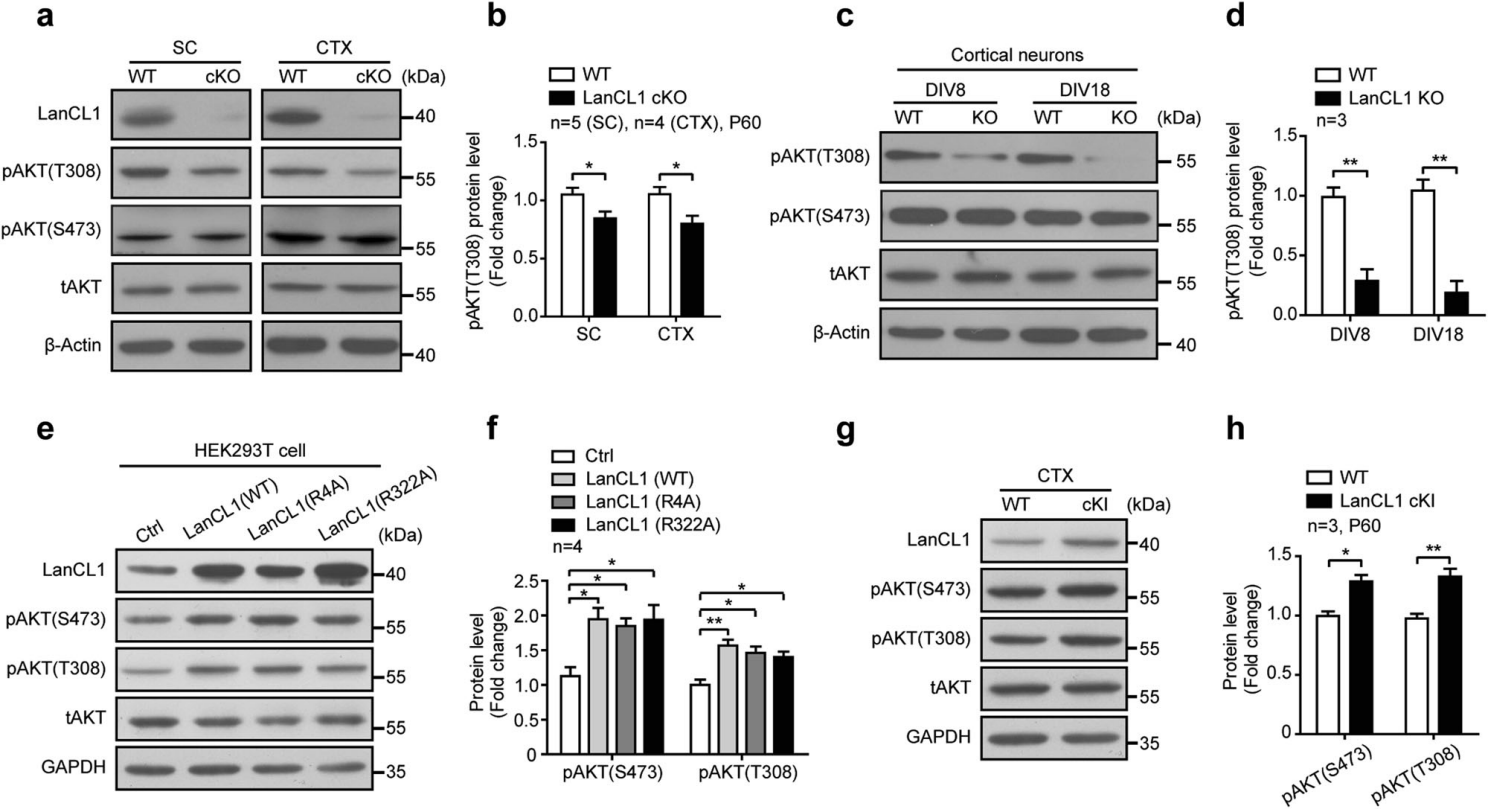
LanCL1 is a positive regulator of AKT activity. **a** Spinal cord and cortex lysates from LanCL1 cKO mice and wild-type (WT) littermates were used in western blots to compare the levels of pAKT(T308) and pAKT(S473). **b** Quantifications showing a reduction in the level of pAKT (T308) in the spinal cord and cortex of LanCL1 cKO mice at P60. Data represent mean ± SEM, spinal cord *n* = 5 mice per genotype, cortex *n* = 4 mice per genotype, *p *<* 0.05, by two-tailed unpaired Student’s *t* test. **c** Lysates from DIV 8 and DIV 18 cortical neurons generated from wild-type or LanCL1-deficient mice were used in western blots to compare the levels of pAKT(T308) and pAKT(S473). **d** Quantifications showing a reduction in the level of pAKT (T308) in cultured LanCL1-deficient cortical neurons at DIV 8 and DIV 18. Data represent mean ± SEM, *n* = 3 mice per genotype per timepoint, **p *<* 0.01, by two-tailed unpaired Student’s *t* test. Immunoblotting (**e**) and quantifications (**f**) show increases in the levels of pAKT(T308) and pAKT(S473) in LanCL1 plasmids (WT or point mutants)-overexpressing HEK293T cells. Data represent mean ± SEM of four independent experiments, *p *<* 0.05, **p *<* 0.01, one-way ANOVA followed by Tukey’s post hoc test. Immunoblotting (**g**) and quantifications (**h**) show increases in the levels of pAKT (T308) and pAKT (S473) in the cortex of P60 LanCL1 cKI mice. Data represent mean ± SEM, *n* = 3 mice per genotype, *p *<* 0.05, **p *<* 0.01, by two-tailed unpaired Student’s *t* test. Quantifications of pAKT (S473) and pAKT (T308) normalized to total AKT

**Fig. 6:**
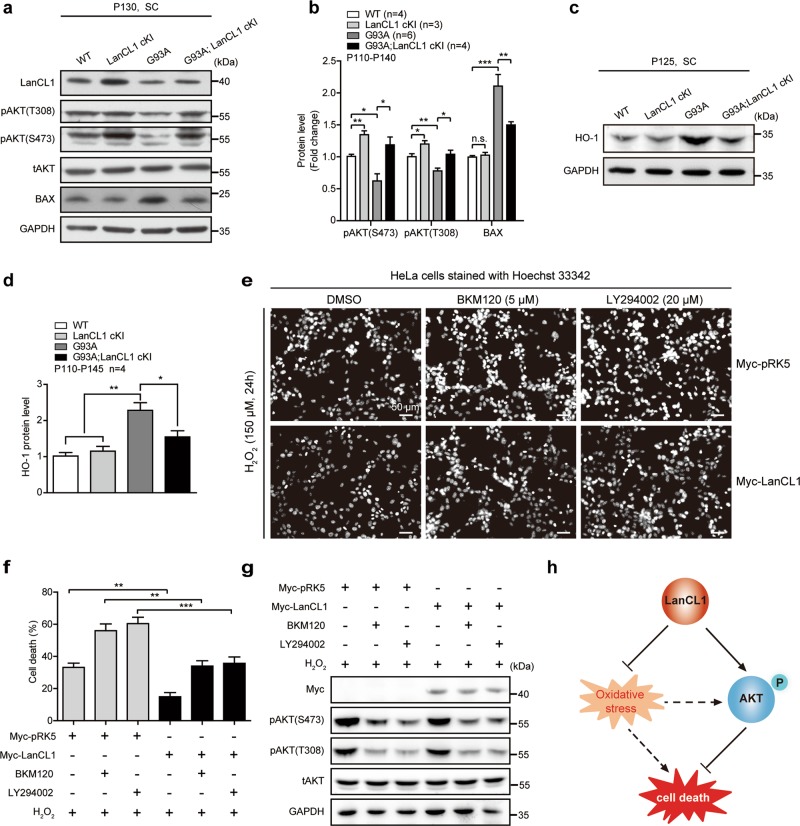
LanCL1 restores impaired AKT activity and mitigates oxidative stress in ALS mice. Immunoblotting (**a**) and quantifications (**b**) of pAKT (S473), pAKT (T308), and BAX in the lumbar spinal cord (L3–L5) of indicated mice. Data represent mean ± SEM; WT *n* = 4 mice, LanCL1 cKI *n* = 3 mice, G93A *n* = 6 mice, G93A; LanCL1 cKI *n* = 4 mice; *p *<* 0.05, **p *<* 0.01, ***p *<* 0.001; n.s. nonsignificant; one-way ANOVA followed by Tukey’s post hoc test. Immunoblotting (**c**) and quantifications (**d**) of HO-1 in the lumbar spinal cord (L3–L5) of indicated mice. Data represent mean ± SEM, *n* = 4 mice per genotype, *p *<* 0.05, **p *<* 0.01, one-way ANOVA followed by Tukey’s post hoc test. **e** HeLa cells were transfected with 2 μg of Myc-pRK5 vector or Myc-tagged LanCL1. Twenty four hours later cells were pretreated with either dimethyl sulfoxide (DMSO), BKM120 (5 μM), or LY294002 (20 μM) for 30 min prior to addition of 150 μM H_2_O_2_. Apoptosis assay was performed by Hoechst 33342 staining 24 h after the H_2_O_2_ treatment. **f** The percentage of apoptotic HeLa cells with fragmented or condensed nuclei was determined by Hoechst 33342 staining. At least 1000 cells per condition were scored for Hoechst 33342 staining. Data represent mean ± SEM of four independent experiments, **p *<* 0.01, ***p *<* 0.001, two-way ANOVA followed by Bonferroni post hoc test. **g** Immunoblotting showing that the PI3K inhibitors (BKM120 and LY294002) effectively blocked AKT activation in response to H_2_O_2_ treatment in HeLa cells. **h** Diagram illustrating that LanCL1 regulates cell survival through mitigating oxidative stress and enhancing AKT phosphorylation

Our previous work demonstrated that LanCL1’s natural function protects neurons through antioxidant defense; deletion of LanCL1 leads to oxidative damage to neurons in the cortex [26]. In the course of examining the mechanisms by which loss of LanCL1 causes neurodegeneration, we found that AKT activation is impaired as a result of LanCL1 deletion. AKT, a serine-threonine kinase, can function to either reduce or prevent cellular destruction from oxidants and plays a critical role in neuronal survival [42, 43]. The activity of AKT is regulated by the phosphorylation of two sites—Thr308 in the catalytic loop by PDK1 and Ser473 in the hydrophobic motif by mTORC2 [44]. We found that loss of LanCL1 significantly decreased the phosphorylation of AKT at Thr308 consistently in the spinal cord and cortex, but to a modest degree at Ser473 and did not reach the level of statistical significance (Fig. 5a, b, Supplementary Fig. 3a). Similar results on AKT phosphorylation status were obtained by the study of cortical neuron cultures derived from WT and LanCL1 KO mice (Fig. 5c, d, Supplementary Fig. 3b). We examined the upstream kinase activity of pAKT(T308)—PDK1, and found that pPDK1(S241) was upregulated in LanCL1 cKO tissues, suggesting that LanCL1 does not regulate AKT phosphorylation through its kinase PDK1 (Supplementary Fig. 3c, d). To examine the role of LanCL1 in the regulation of AKT phosphorylation, we transfected LanCL1 in HEK293T cells and assessed its effect on AKT phosphorylation. LanCL1 transfection elevated AKT phosphorylation at both Thr308 and Ser473 sites (Fig. 5e, f). The effect of LanCL1 on AKT phosphorylation appears to be independent of its GSH-binding capacity because LanCL1 point mutants (R4A and R322A) that failed to bind GSH [25] showed similar effect on AKT phosphorylation as WT LanCL1 (Fig. 5e, f). Consistent with results obtained by in vitro transfection of LanCL1, expression of LanCL1 transgene in neural cells increased AKT phosphorylation at Thr308 and Ser473 in the cortex and spinal cord of LanCL1 cKI mice (Figs. 5g, h and 6a, b). Together, these results indicate that LanCL1 is a positive regulator of AKT activity.

### LanCL1 exerts neuronal protective function through mitigating oxidative stress and enhancing AKT activity

Deregulation of AKT activity is closely associated with neurodegenerative diseases such as ALS [20, 45, 46]. To determine whether LanCL1 regulated AKT activity plays a role in conferring neuroprotection in ALS mice, we evaluated AKT phosphorylation/activation in the G93A; LanCL1 cKI mice, G93A mice and control littermates. LanCL1 overexpression restored AKT activity which is impaired in G93A mice (Fig. 6a, b). Accordingly, expression of LanCL1 transgene blocked the induction of the proapoptotic Bcl-2 family member BAX (Fig. 6a, b). Consistent with the notion that LanCL1 possess antioxidant function, the oxidative damage in the spinal cord of the G93A mice was ameliorated by LanCL1 transgene. G93A mice exhibited a significant increase in HO-1 (also known as HMOX1) expression, a marker of oxidative stress [13], compared with WT and LanCL1 cKI mice (Fig. 6c, d). However, LanCL1 transgene reduced HO-1 by ~23% in G93A; LanCL1 cKI mice (Fig. 6c, d).

LanCL1 regulated AKT activity is relevant to cell survival, because inhibiting activation of PI3K/AKT pathway with the PI3K inhibitor (BKM120 or LY294002) altered survival of H_2_O_2_-treated cells (Supplementary Fig. 4a, Fig. 6e, f) and the protective effect of LanCL1 overexpression against H_2_O_2_-induced cell death was compromised by inhibiting AKT activity (Fig. 6e–g). However, LanCL1’s protective effect was not completely abrogated by blocking AKT activation, as more LanCL1-overexpressing cells survived when the cytotoxic effect of H_2_O_2_ was potentiated by inhibiting AKT activity (Fig. 6e–g). This finding argues for an AKT-independent role of LanCL1 in the control of cell survival, which is consistent with its ROS scavenging function as previously described [26]. We are beginning to understand the interplay between LanCL1, oxidative stress and AKT signaling. AKT was activated by H_2_O_2_ treatment in a concentration- and time-dependent manner (Supplementary Fig. 4b–g), consistent with previous studies [47, 48]. When the concentration of H_2_O_2_ was low (less than 100 μM), LanCL1 transgene was able to potentiate H_2_O_2_ induced AKT phosphorylation (Supplementary Fig. 4b–d). However, AKT phosphorylation seemed to be already at its maximal level with H_2_O_2_ at 150 μM (Supplementary Fig. 4b–g). These results suggest a dynamic relationship among oxidative stress, LanCL1, and AKT activation that needs to be explored in the future studies. Taken together, our findings suggest a model as shown in Fig. 6h, which depicts that LanCL1 could not only reduce oxidative stress as a ROS scavenger, but also function as an AKT activator in the control of cell survival (Fig. 6h).

## Discussion

Our previous work identifies LanCL1 as an antioxidant defense gene which is predominantly expressed in neurons and possesses ROS scavenging properties like GSTs [26]. Here, we found that CNS-specific LanCL1 transgene delays disease onset, decelerates disease progression, and prolongs lifespan in the SOD1^G93A^ mouse model of ALS. These disease-modifying effects of LanCL1 transgene might be a direct result of its role in protecting the MNs in the spinal cord from degeneration in the ALS model, as the age-dependent loss of spinal MNs is significantly delayed. These results demonstrate the neuroprotective effect of LanCL1 transgene in ALS mouse model and support the translational potential of LanCL1.

Since the identification of mutations in the SOD1 gene as an important cause of familial ALS [3] and the generation of the SOD1 mouse model of ALS [4], genetic modifications on SOD1 mouse model have been described, mostly focusing on the putative disease mechanisms, such as oxidative stress, excitotoxicity, axonal transport defects, mitochondrial dysfunction, and apoptosis [49]. Unfortunately, most genetic approaches have failed to extend survival and in some cases have even hastened disease progression in the rodent models of ALS [49, 50]. For example, Genetic elevation or elimination of glutathione peroxidase 1 (GPx1) impacted on neither onset nor survival in SOD1^G93A^ mice [51]. Herein, CNS-specific expression of LanCL1 transgene results in great survival elongation (~15%), delayed disease onset (13–16%), and reduced MN loss (27–34%). It is probable that besides antioxidant defense, other functions of LanCL1 are also important for neuronal protection. Of note, since the Nestin-cre drives LanCL1 overexpression also in glial cells, we cannot rule out the contribution of these glial cells to the overall effects of LanCL1 transgene, as these cells are known to contribute to ALS pathogenesis [52–54].

The AKT pathway is well known to regulate neuronal survival, and plays a vital role in neurodegenerative diseases, such as ALS [20, 46, 55, 56]. Multiple studies have reported impaired AKT-mediated prosurvival signaling pathway in ALS mouse models and in patients as well [20, 45, 55]. In this study, we identify LanCL1 as a novel activator of AKT kinase. Genetic deletion of LanCL1 reduces AKT phosphorylation in the brain and spinal cord, whereas the expression of LanCL1 transgene in vitro and in vivo elevates AKT phosphorylation. The AKT activity regulated by LanCL1 contributes to cell protection, as inhibiting activation of AKT pathway altered survival of H_2_O_2_-treated cells. Here, the neuroprotective effect of LanCL1 transgene in the ALS mouse model could be the combined effect of LanCL1 as a ROS scavenger and an activator of AKT activity. Besides, the relationship between AKT signaling and oxidative stress is complex, since AKT not only modulates oxidative stress but also is affected by ROS activation [48, 57]. Thus, there is an intriguing link between LanCL1, oxidative stress and AKT signaling (Fig. 6h). Additional studies are needed to explore the molecular mechanisms by which LanCL1 regulates the AKT activity. We found that the upstream kinase of AKT–PDK1 activity was upregulated in LanCL1 cKO tissues, suggesting that LanCL1 does not function through activating PDK1. Activating AKT might be a conserved function of LanC-like (LanCL) protein family. LanCL2, a homology of LanCL1, was recently reported to promote AKT activity via direct physical interactions with both the AKT kinase and the substrate [58].

Hensley et al. propose that LanCL1 catalyzes the addition of the Cys of glutathione to protein- or peptide-bound dehydroalanine to form lanthionine, analogous to the reaction catalyzed by LanC in bacteria, and then converted to lanthionine ketimine (LK) in the mammalian brain [59]. Surprisingly, a cell-permeable ester derivative of LK (LKE) appears to possess antioxidative stress and antiapoptotic function in NSC-34 MN-like cells and increase survival in SOD1^G93A^ transgenic mice [59, 60]. Even though the hypothesis that LanCL1 is involved in LK biosynthesis remains controversial at present [61], it is possible that other functional link between LanCL1 and LK may exist and therefore contributes to neuronal protection.

LanCL1 is enriched in neurons and dynamically regulated by growth factors, neuronal activity, and oxidative stress [26]. These facts indicate that LanCL1 expression may be dysregulated in neurodegenerative diseases, such as ALS. The endogenous LanCL1 expression is upregulated at late presymptomatic stage (P75) in the spinal cord of ALS mice, which suggests a protective or compensatory function in the time frame prior to protein oxidation, neuroinflammatory acceleration, and MN death, as proposed by Chung CH et al. [27]. However, due to unknown transcriptional or post-transcriptional alternations, LanCL1 expression is progressively decreased as the disease progresses. Thus, further research is needed to focus on the disease progression-dependent expression of LanCL1 in ALS mice.

In conclusion, this study demonstrates that LanCL1 confers neuronal protection in a disease setting and establish that LanCL1 is a new therapeutic target for ALS. It also supports the notion that mitigating oxidative stress combined with boosting AKT activity represents a promising therapeutic approach to treating ALS and related neurodegeneration diseases.

## Supplementary information

Supplementary Information

Supplementary video 1

## References

[1] Hardiman O, Al-Chalabi A, Chio A, Corr EM, Logroscino G, Robberecht W (2017). Amyotrophic lateral sclerosis. Nat Rev Dis Primers.

[2] Robberecht W, Philips T (2013). The changing scene of amyotrophic lateral sclerosis. Nat Rev Neurosci.

[3] Rosen DR, Siddique T, Patterson D, Figlewicz DA, Sapp P, Hentati A (1993). Mutations in Cu/Zn superoxide dismutase gene are associated with familial amyotrophic lateral sclerosis. Nature.

[4] Gurney ME, Pu H, Chiu AY, Dal Canto MC, Polchow CY, Alexander DD (1994). Motor neuron degeneration in mice that express a human Cu,Zn superoxide dismutase mutation. Science.

[5] Logroscino G, Traynor BJ, Hardiman O, Chio A, Mitchell D, Swingler RJ (2010). Incidence of amyotrophic lateral sclerosis in Europe. J Neurol Neurosurg Psychiatry.

[6] Heiman-Patterson TD, Deitch JS, Blankenhorn EP, Erwin KL, Perreault MJ, Alexander BK (2005). Background and gender effects on survival in the TgN(SOD1-G93A)1Gur mouse model of ALS. J Neurological Sci.

[7] Bensimon G, Lacomblez L, Meininger V (1994). A controlled trial of riluzole in amyotrophic lateral sclerosis. ALS/Riluzole Study Group. New Engl J Med.

[8] Yoshino H, Kimura A (2006). Investigation of the therapeutic effects of edaravone, a free radical scavenger, on amyotrophic lateral sclerosis (Phase II study). Amyotroph Lateral Scler.

[9] Ferrante RJ, Browne SE, Shinobu LA, Bowling AC, Baik MJ, MacGarvey U (1997). Evidence of increased oxidative damage in both sporadic and familial amyotrophic lateral sclerosis. J Neurochem.

[10] Beal MF, Ferrante RJ, Browne SE, Matthews RT, Kowall NW, Brown RH (1997). Increased 3-nitrotyrosine in both sporadic and familial amyotrophic lateral sclerosis. Ann Neurol.

[11] Shibata N, Nagai R, Uchida K, Horiuchi S, Yamada S, Hirano A, et al. Morphological evidence for lipid peroxidation and protein glycoxidation in spinal cords from sporadic amyotrophic lateral sclerosis patients. Brain research. 2001;917:97–104. 10.1016/s0006-8993(01)02926-2 11602233

[12] Bogdanov M, Brown RH, Matson W, Smart R, Hayden D, O’Donnell H, et al. Increased oxidative damage to DNA in ALS patients. Free Radic Biol Med. 2000;29:652–8. 10.1016/s0891-5849(00)00349-x 11033417

[13] Ferrante RJ, Shinobu LA, Schulz JB, Matthews RT, Thomas CE, Kowall NW, et al. Increased 3-nitrotyrosine and oxidative damage in mice with a human copper/zinc superoxide dismutase mutation. Ann Neurol. 1997;42:326–34. 10.1002/ana.410420309 9307254

[14] Andrus PK, Fleck TJ, Gurney ME, Hall ED (1998). Protein oxidative damage in a transgenic mouse model of familial amyotrophic lateral sclerosis. J Neurochem.

[15] Liu R, Althaus JS, Ellerbrock BR, Becker DA, Gurney ME. Enhanced oxygen radical production in a transgenic mouse model of familial amyotrophic lateral sclerosis. Ann Neurol. 1998;44:763–70. 10.1002/ana.410440510 9818932

[16] Barber SC, Mead RJ, Shaw PJ. Oxidative stress in ALS: a mechanism of neurodegeneration and a therapeutic target. Biochim Biophys Acta. 2006;1762:1051–67. 10.1016/j.bbadis.2006.03.008 16713195

[17] Ilieva H, Polymenidou M, Cleveland DW (2009). Non–cell autonomous toxicity in neurodegenerative disorders: ALS and beyond. J Cell Biol.

[18] Alexianu ME, Kozovska M, Appel SH. Immune reactivity in a mouse model of familial ALS correlates with disease progression. Neurology. 2001;57:1282–9. 10.1212/wnl.57.7.1282 11591849

[19] Ferraiuolo L, Kirby J, Grierson AJ, Sendtner M, Shaw PJ (2011). Molecular pathways of motor neuron injury in amyotrophic lateral sclerosis. Nat Rev Neurol.

[20] Kirby J, Ning K, Ferraiuolo L, Heath PR, Ismail A, Kuo SW (2011). Phosphatase and tensin homologue/protein kinase B pathway linked to motor neuron survival in human superoxide dismutase 1-related amyotrophic lateral sclerosis. Brain.

[21] Bauer H, Mayer H, Marchler-Bauer A, Salzer U, Prohaska R (2000). Characterization of p40/GPR69A as a peripheral membrane protein related to the lantibiotic synthetase component C. Biochem Biophys Res Commun.

[22] Chatterjee C, Paul M, Xie L, van der Donk WA (2005). Biosynthesis and mode of action of lantibiotics. Chem Rev.

[23] Mayer H, Salzer U, Breuss J, Ziegler S, Marchler-Bauer A, Prohaska R (1998). Isolation, molecular characterization, and tissue-specific expression of a novel putative G protein-coupled receptor. Biochim Biophys Acta.

[24] Mayer H, Bauer H, Breuss J, Ziegler S, Prohaska R (2001). Characterization of rat LANCL1, a novel member of the lanthionine synthetase C-like protein family, highly expressed in testis and brain. Gene..

[25] Zhang W, Wang L, Liu Y, Xu J, Zhu G, Cang H (2009). Structure of human lanthionine synthetase C-like protein 1 and its interaction with Eps8 and glutathione. Genes Dev.

[26] Huang C, Chen M, Pang D, Bi D, Zou Y, Xia X (2014). Developmental and activity-dependent expression of LanCL1 confers antioxidant activity required for neuronal survival. Dev Cell.

[27] Chung CH, Kurien BT, Mehta P, Mhatre M, Mou S, Pye QN (2007). Identification of lanthionine synthase C-like protein-1 as a prominent glutathione binding protein expressed in the mammalian central nervous system. Biochemistry..

[28] Tronche F, Kellendonk C, Kretz O, Gass P, Anlag K, Orban PC (1999). Disruption of the glucocorticoid receptor gene in the nervous system results in reduced anxiety. Nat Genet.

[29] Boillee S, Yamanaka K, Lobsiger CS, Copeland NG, Jenkins NA, Kassiotis G (2006). Onset and progression in inherited ALS determined by motor neurons and microglia. Science.

[30] Mironets E, Osei-Owusu P, Bracchi-Ricard V, Fischer R, Owens EA, Ricard J (2018). Soluble TNFalpha signaling within the spinal cord contributes to the development of autonomic dysreflexia and ensuing vascular and immune dysfunction after spinal cord injury. J Neurosci.

[31] Cai M, Choi SM, Yang EJ. The effects of bee venom acupuncture on the central nervous system and muscle in an animal hSOD1G93A mutant. Toxins. 2015;7:846–58. 10.3390/toxins7030846 PMC4379529 25781653

[32] Guo Y, Zhang Y, Wen D, Duan W, An T, Shi P (2013). The modest impact of transcription factor Nrf2 on the course of disease in an ALS animal model. Lab Investig.

[33] Kaech S, Banker G. Culturing hippocampal neurons. Nat Protoc. 2006;1:2406–15. 10.1038/nprot.2006.356 17406484

[34] Feng C, Luo T, Zhang S, Liu K, Zhang Y, Luo Y, et al. Lycopene protects human SHSY5Y neuroblastoma cells against hydrogen peroxideinduced death via inhibition of oxidative stress and mitochondriaassociated apoptotic pathways. Molecular medicine reports. 2016;13:4205–14. 10.3892/mmr.2016.5056 PMC4838073 27035331

[35] Ouali Alami N, Schurr C, Olde Heuvel F, Tang L, Li Q, Tasdogan A, et al. NF-kappaB activation in astrocytes drives a stage-specific beneficial neuroimmunological response in ALS. EMBO J. 2018;37:e98697. 10.15252/embj.201798697 PMC6092622 29875132

[36] Miyoshi S, Tezuka T, Arimura S, Tomono T, Okada T, Yamanashi Y. DOK7 gene therapy enhances motor activity and life span in ALS model mice. EMBO Mol Med. 2017;9:880–9. 10.15252/emmm.201607298 PMC5494517 28490573

[37] Lee JK, Shin JH, Hwang SG, Gwag BJ, McKee AC, Lee J (2013). MST1 functions as a key modulator of neurodegeneration in a mouse model of ALS. PNAS.

[38] Ludolph AC, Bendotti C, Blaugrund E, Hengerer B, Loffler JP, Martin J (2007). Guidelines for the preclinical in vivo evaluation of pharmacological active drugs for ALS/MND: report on the 142nd ENMC international workshop. Amyotroph Lateral Scler.

[39] Tsang YM, Chiong F, Kuznetsov D, Kasarskis E, Geula C (2000). Motor neurons are rich in non-phosphorylated neurofilaments: cross-species comparison and alterations in ALS. Brain Res.

[40] Nystrom T (2005). Role of oxidative carbonylation in protein quality control and senescence. EMBO J.

[41] Esterbauer H, Zollner H, Lang J (1985). Metabolism of the lipid peroxidation product 4-hydroxynonenal by isolated hepatocytes and by liver cytosolic fractions. Biochemical J.

[42] Dudek H, Datta SR, Franke TF, Birnbaum MJ, Yao R, Cooper GM, et al. Regulation of neuronal survival by the serine-threonine protein kinase Akt. Science. 1997;275:661–5. 10.1126/science.275.5300.661 9005851

[43] Yamaguchi A, Tamatani M, Matsuzaki H, Namikawa K, Kiyama H, Vitek MP (2001). Akt activation protects hippocampal neurons from apoptosis by inhibiting transcriptional activity ofp53. J Biol Chem.

[44] Manning BD, Toker A. AKT/PKB signaling: navigating the network. Cell. 2017;169:381–405. 10.1016/j.cell.2017.04.001 PMC5546324 28431241

[45] Koh SH, Kwon H, Kim KS, Kim J, Kim MH, Yu HJ (2004). Epigallocatechin gallate prevents oxidative-stress-induced death of mutant Cu/Zn-superoxide dismutase (G93A) motoneuron cells by alteration of cell survival and death signals. Toxicology..

[46] Yin X, Ren M, Jiang H, Cui S, Wang S, Jiang H (2015). Downregulated AEG-1 together with inhibited PI3K/Akt pathway is associated with reduced viability of motor neurons in an ALS model. Mol Cell Neurosci.

[47] Sadidi M, Lentz SI, Feldman EL (2009). Hydrogen peroxide-induced Akt phosphorylation regulates Bax activation. Biochimie..

[48] Wang X, McCullough KD, Franke TF, Holbrook NJ (2000). Epidermal growth factor receptor-dependent Akt activation by oxidative stress enhances cell survival. J Biol Chem.

[49] Turner BJ, Talbot K (2008). Transgenics, toxicity and therapeutics in rodent models of mutant SOD1-mediated familial ALS. Prog Neurobiol.

[50] Riboldi G, Nizzardo M, Simone C, Falcone M, Bresolin N, Comi GP, et al. ALS genetic modifiers that increase survival of SOD1 mice and are suitable for therapeutic development. Prog Neurobiol. 2011;95:133-48. 10.1016/j.pneurobio.2011.07.009 21816207

[51] Cudkowicz ME, Pastusza KA, Sapp PC, Mathews RK, Leahy J, Pasinelli P, et al. Survival in transgenic ALS mice does not vary with CNS glutathione peroxidase activity. Neurology. 2002;59:729–34. 10.1212/wnl.59.5.729 12221165

[52] Kang SH, Li Y, Fukaya M, Lorenzini I, Cleveland DW, Ostrow LW (2013). Degeneration and impaired regeneration of gray matter oligodendrocytes in amyotrophic lateral sclerosis. Nat Neurosci.

[53] Frakes AE, Ferraiuolo L, Haidet-Phillips AM, Schmelzer L, Braun L, Miranda CJ (2014). Microglia induce motor neuron death via the classical NF-kappa B pathway in amyotrophic lateral sclerosis. Neuron.

[54] Qian K, Huang H, Peterson A, Hu B, Maragakis NJ, Ming GL, et al. Sporadic ALS astrocytes induce neuronal degeneration in vivo. Stem Cell Rep. 2017;8:843–55. 10.1016/j.stemcr.2017.03.003 PMC5390239 28366455

[55] Dewil M, Lambrechts D, Sciot R, Shaw PJ, Ince PG, Robberecht W, et al. Vascular endothelial growth factor counteracts the loss of phospho-Akt preceding motor neurone degeneration in amyotrophic lateral sclerosis. Neuropathol Appl Neurobiol. 2007;33:499–509. 10.1111/j.1365-2990.2007.00850.x 17854437

[56] Peviani M, Tortarolo M, Battaglia E, Piva R, Bendotti C (2014). Specific induction of Akt3 in spinal cord motor neurons is neuroprotective in a mouse model of familial amyotrophic lateral sclerosis. Mol Neurobiol.

[57] Zhang J, Wang X, Vikash V, Ye Q, Wu D, Liu Y, et al. ROS and ROS-Mediated Cellular Signaling. Oxid Med Cell Longev. 2016;2016:4350965. 10.1155/2016/4350965 PMC4779832 26998193

[58] Zeng M, van der Donk WA, Chen J (2014). Lanthionine synthetase C-like protein 2 (LanCL2) is a novel regulator of Akt. Mol Biol Cell.

[59] Hensley K, Venkova K, Christov A (2010). Emerging biological importance of central nervous system lanthionines. Molecules..

[60] Nada SE, Tulsulkar J, Raghavan A, Hensley K, Shah ZA (2012). A derivative of the CRMP2 binding compound lanthionine ketimine provides neuroprotection in a mouse model of cerebral ischemia. Neurochem Int.

[61] He C, Zeng M, Dutta D, Koh TH, Chen J, van der Donk WA (2017). LanCL proteins are not Involved in Lanthionine Synthesis in Mammals. Sci Rep.

