# Supplementary material for: LanCL1 promotes motor neuron survival and extends the lifespan of amyotrophic lateral sclerosis mice
Source: Cell Death Differ. 2019 Sep 30;27(4):1369–82. doi: 10.1038/s41418-019-0422-6 (PMC7206132; doi:10.1038/s41418-019-0422-6)
Supplement: Supplementary file 1 — Supplementary Information [file 41418_2019_422_MOESM1_ESM.docx]

**Supplementary Information**

**LanCL1 Promotes Motor Neuron Survival and Extends the Lifespan of Amyotrophic Lateral Sclerosis Mice**

Honglin Tan, Mina Chen, Dejiang Pang, Xiaoqiang Xia, Chongyangzi Du, Wanchun Yang, Yiyuan Cui, Chao Huang, Wanxiang Jiang, Dandan Bi, Chunyu Li, Huifang Shang, Paul F. Worley, Bo Xiao

**Supplementary figures** **and figure legends**


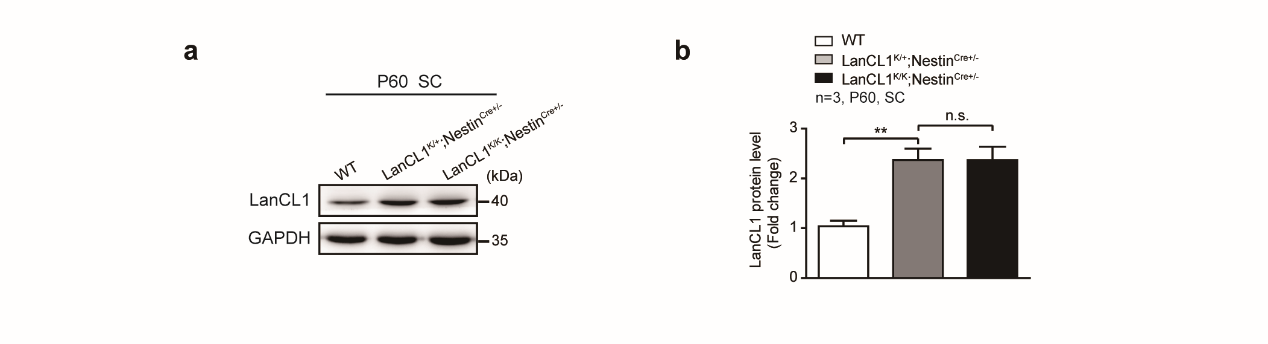


**Figure S1. Expression of LanCL1 transgene in the spinal cord of LanCL1^K/+^;Nestin^Cre+/-^ and LanCL1^K/K^;Nestin^Cre+/-^ mice. a,b** Immunoblotting (**a**) and quantifications (**b**) of LanCL1 in the lumbar spinal cord (L3-L5) of WT, LanCL1^K/+^;Nestin^Cre+/-^ and LanCL1^K/K^;Nestin^Cre+/-^ mice at P60. Data represent mean ± SEM, n=3 mice per genotype, ***p* < 0.01, n.s., non-significant, one-way ANOVA followed by Tukey’s post hoc test.


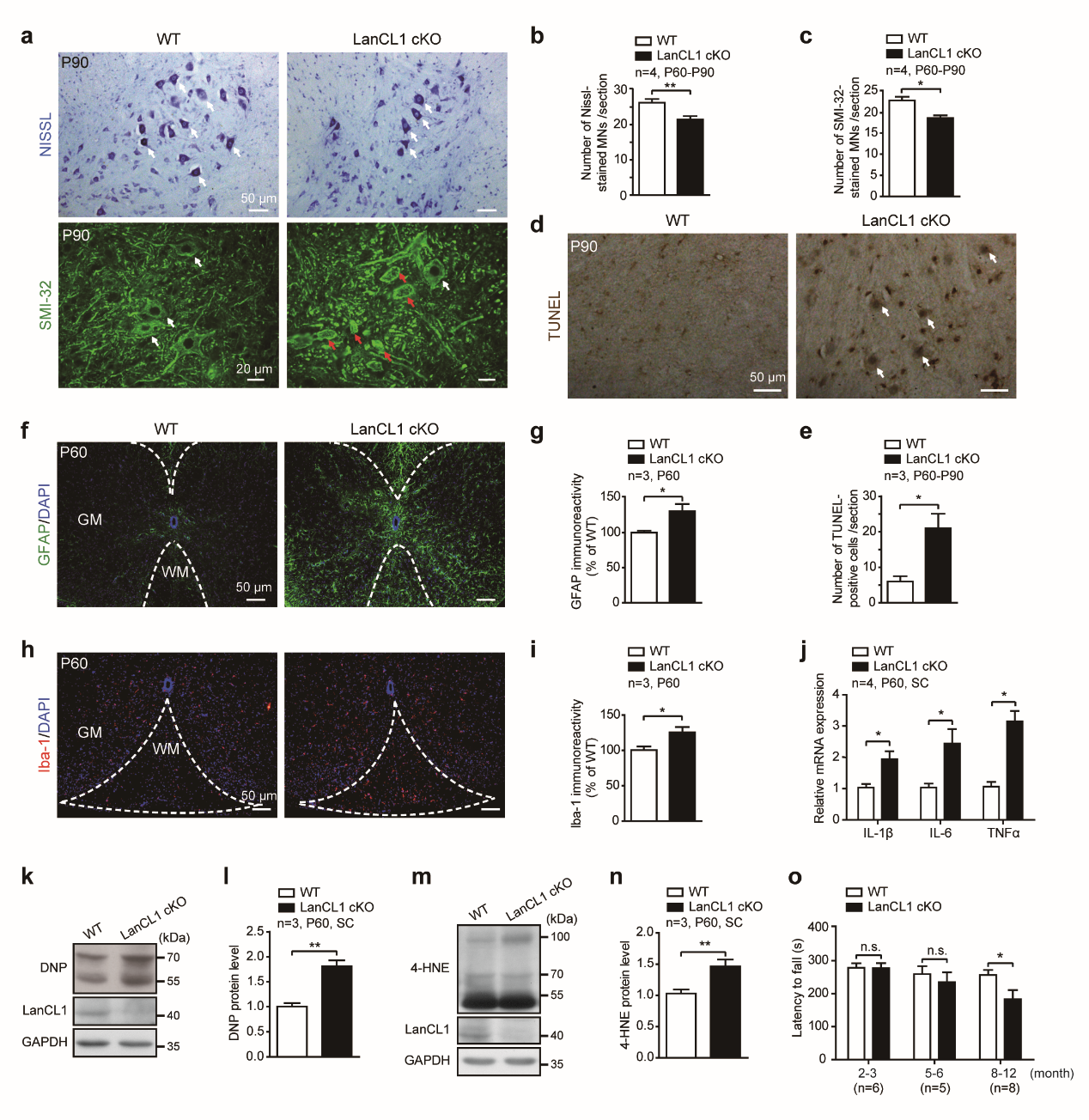


**Figure S2. CNS-specific loss of LanCL1 leads to MN degeneration, neuroinflammation and oxidative damage in the spinal cord. a** Representative images of spinal ventral horn from P90 WT and LanCL1 cKO mice, stained with Nissl (the upper panel) or SMI-32 (the lower panel). White arrows indicate normal MNs, whereas red arrows indicate degenerative MNs. **b,c** Quantification of Nissl^+^ MNs (**b**) and SMI-32^+^ MNs (**c**) per spinal cord section in WT and LanCL1 cKO mice at P60 to P90. Data represent mean ± SEM, n=4 mice per genotype, **p* < 0.05, ***p* < 0.01, by Student’s unpaired *t*-test. **d** TUNEL staining showing an increase of cell death (indicated by white arrows) in the spinal ventral horn of LanCL1 cKO mice at P90. **e** Quantification of TUNEL^+^ cells per spinal cord section at P60 to P90. Data represent mean ± SEM, n=3 mice per genotype, **p* < 0.05, by Student’s unpaired *t*-test. **f,g** Representative images (**f**) and quantifications (**g**) of astrocyte stained for GFAP (green) and DAPI (blue) in the spinal cord of WT and LanCL1 cKO mice at P60. GW, gray matter, WM, white matter. Data represent mean ± SEM, n=3 mice per group, **p* < 0.05, by Student’s unpaired *t*-test. **h,i** Representative images (**h**) and quantifications (**i**) of microglia stained for Iba-1 (red) and DAPI (blue) in the spinal cord of WT and LanCL1 cKO mice at P60. Data represent mean ± SEM, n=3 mice per group, **p* < 0.05, by Student’s unpaired *t*-test. **j** Expression of *IL-1β, IL-6* and *TNF-α* in the spinal cord of WT and LanCL1 cKO mice assayed by quantitative real-time PCR. Data represent mean ± SEM, n=4 mice per group, **p* < 0.05, by Student’s unpaired *t*-test. **k,l** Immunoblotting (**k**) and quantifications (**l**) showing an increase of DNP in the spinal cord of LanCL1 cKO mice at P60. Data represent mean ± SEM, n=3 mice per group, ***p* < 0.01, by Student’s unpaired *t*-test. **m,n** Immunoblots (**m**) and quantifications (**n**) showing an increase in the level of 4-HNE in the spinal cord of LanCL1 cKO mice at P60. Data represent mean ± SEM, n=3 mice per group, ***p* < 0.01, by Student’s unpaired *t*-test. **o** Rotarod tests showing that aged LanCL1 cKO mice (8-12 month) display significant decrease in the average latency to fall. Data represent mean ± SEM, 2-3 month n=6 mice per genotype, 5-6 month n=5 mice per genotype, 8-12 month n=8 mice per genotype, **p* < 0.05, n.s., non-significant, two-way ANOVA followed by Bonferroni post hoc test.


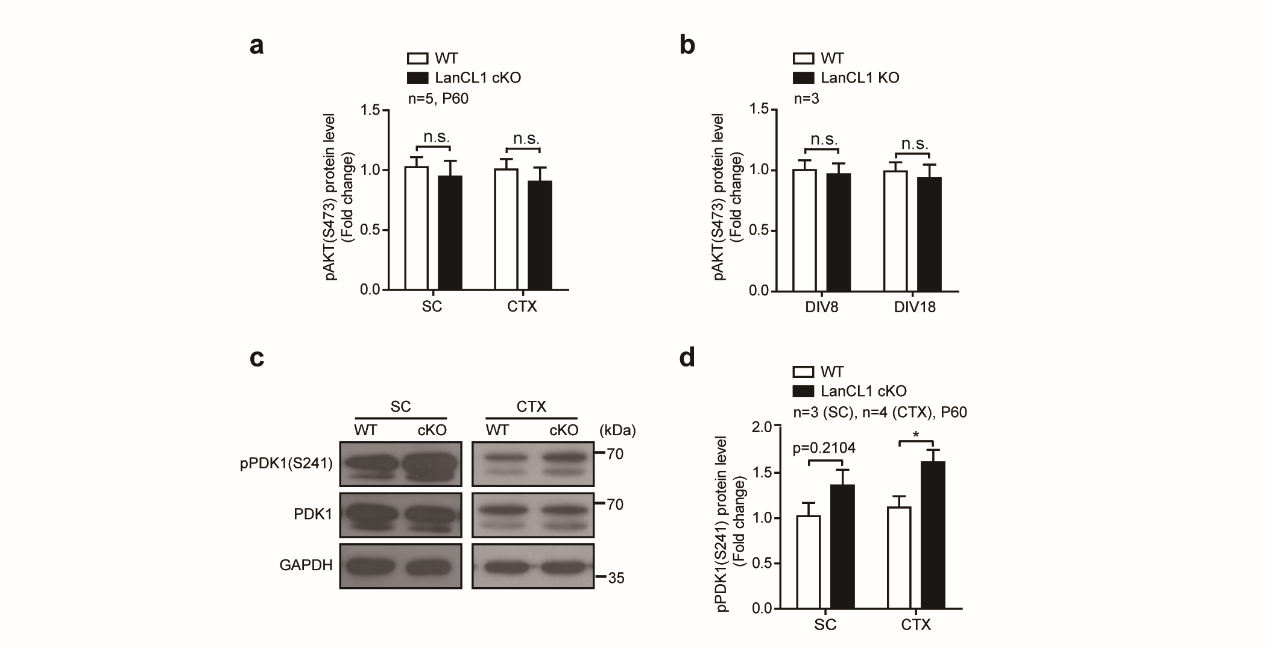


**Figure S3. Effects of LanCL1 deletion on pAKT(S473) and pPDK1(S241). a** Quantifications of pAKT (S473) protein levels in the spinal cord and cortex of WT and LanCL1 cKO mice at P60. Data represent mean ± SEM, spinal cord n=5 mice per genotype, cortex n=5 mice per genotype, n.s., non-significant, by two-tailed unpaired Student’s *t*-test. **b** Quantifications of pAKT (S473) in cultured WT and LanCL1-deficient cortical neurons at DIV 8 and DIV 18. Data represent mean ± SEM, n=3 mice per genotype per timepoint, n.s., non-significant, by two-tailed unpaired Student’s *t*-test. **c,d** Immunoblotting (**c**) and quantifications (**d**) of pPDK1(S241) levels in the spinal cord and cortex of WT and LanCL1 cKO mice at P60. Data represent mean ± SEM, spinal cord n=3 mice per genotype, cortex n=4 mice per genotype, *p*=0.2104 (spinal cord), **p* < 0.05 (cortex), by two-tailed unpaired Student’s *t*-test. Quantifications of pAKT (S473) and pPDK1 (S241) normalized to total AKT and PDK1, respectively.


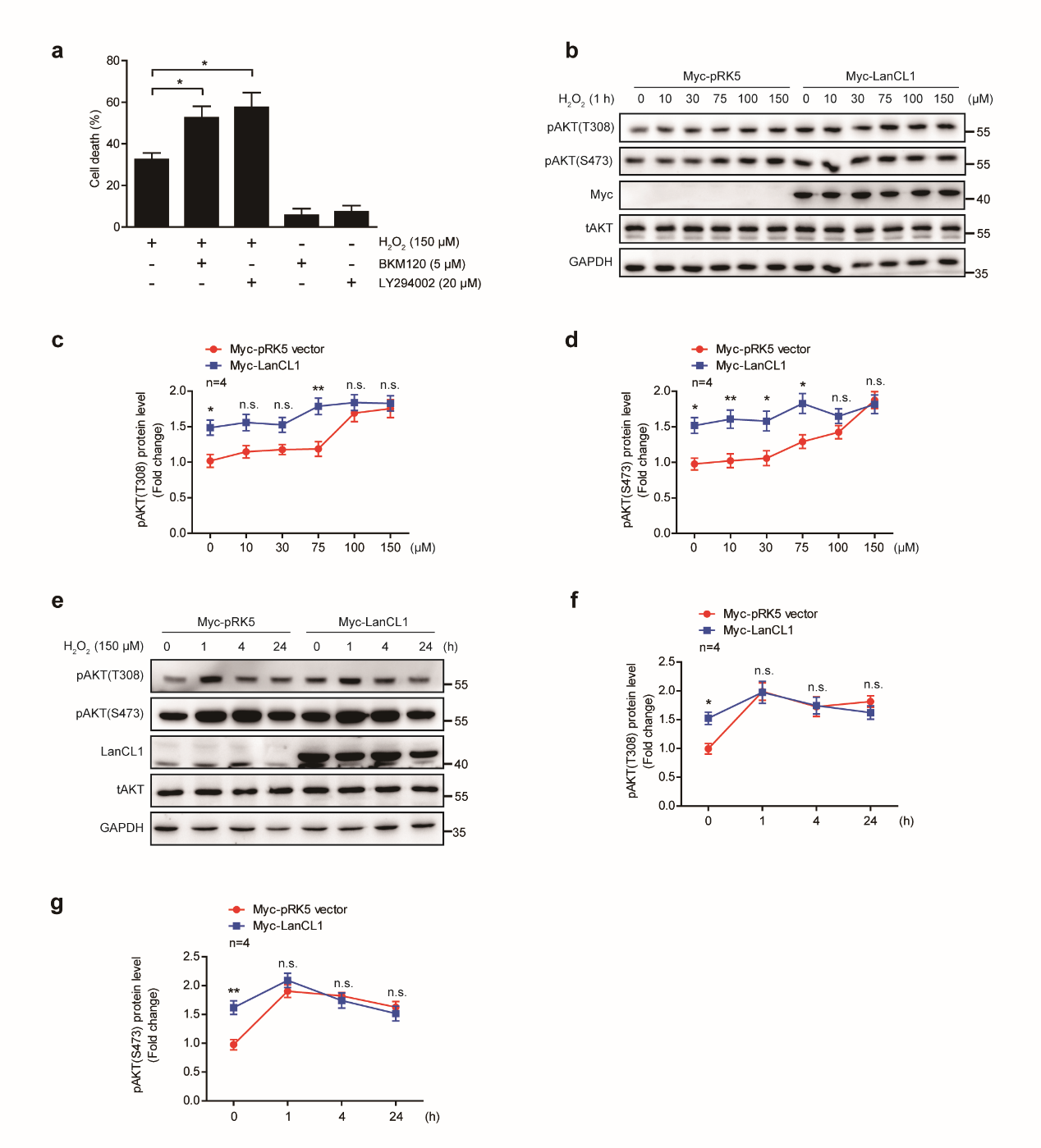


**Figure S4. Pretreatment with PI3K inhibitors enhances H_2_O_2_-induced cell apoptosis in HeLa cells, and AKT is activated by H_2_O_2_ treatment in a concentration- and time-dependent manner. a** HeLa cells were pretreated with either 5 μM BKM120 or 20 μM LY294002 for 30 min prior to addition of 150 μM H_2_O_2_. Apoptosis was assessed by Hoechst 33342 staining 24 hours later. Data represent mean ± SEM of three independent experiments, **p* < 0.05, one-way ANOVA followed by Tukey’s post hoc test. **b** HeLa cells were transfected with 2 μg of Myc-pRK5 vector or Myc-tagged LanCL1. Twenty four hours later cells were treated with different doses of H_2_O_2_. AKT phosphorylation was assayed by immunoblotting 1 hour after H_2_O_2_ treatment. **c,d** Relative pAKT(T308) (**c**) and pAKT(S473) (**d**) levels (normalized to tAKT) were quantified. Data represent mean ± SEM of four independent experiments, **p* < 0.05, ***p* < 0.01, n.s., non-significant, two-way ANOVA followed by Bonferroni post hoc test. **e** HeLa cells were transfected with 2 μg of Myc-pRK5 vector or Myc-tagged LanCL1. Twenty four hours later cells were treated with 150 μM H_2_O_2_ and harvested at various times thereafter. The cell lysates were analyzed by immunoblotting using specific antibodies against pAKT (T308), pAKT(S473), tAKT and LanCL1. **f,g** Relative pAKT(T308) (**f**) and pAKT(S473) (**g**) levels (normalized to tAKT) were quantified. Data represent mean ± SEM of four independent experiments, **p* < 0.05, ***p* < 0.01, n.s., non-significant, two-way ANOVA followed by Bonferroni post hoc test.
